# Measuring health related quality of life (HRQoL) in community and facility-based care settings with the interRAI assessment instruments: development of a crosswalk to HUI3

**DOI:** 10.1007/s11136-018-1800-0

**Published:** 2018-02-12

**Authors:** John P. Hirdes, Julie Bernier, Rochelle Garner, Philippe Finès, Micaela Jantzi

**Affiliations:** 1 School of Public Health and Health Systems, University of Waterloo, Waterloo, ON N2L 3G1 Canada; 2 Health Analysis Division, Statistics Canada, Ottawa, Ontario K1A 0T6 Canada

**Keywords:** InterRAI, HUI3, Nursing homes, Home care, Assessment

## Abstract

**Background:**

Health-related quality of life (HRQoL) measures are of interest because they can be used to describe health of populations and represent a broader health outcome for population health analyses than mortality rates or life expectancy. The most widely used measure of HRQoL for deriving estimates of health-adjusted life expectancy is the Health Utilities Index Mark 3 (HUI3). The HUI3 is available in most national surveys administered by Statistics Canada, and has been used as part of a microsimulation model to examine the impact of neurological conditions over the life course. Persons receiving home care and nursing home services are often not well-represented in these surveys; however, interRAI assessment instruments are now used as part of normal clinical practice in these settings for nine Canadian provinces/territories. Building on previous research that developed a HUI2 crosswalk for the interRAI assessments, the present study examined a new interRAI HRQoL index crosswalked to the HUI3.

**Methods:**

interRAI and survey data were used to examine the distributional properties of global and domain-specific interRAI HRQoL and HUI3 index scores, respectively. Three populations were considered: well-elderly persons not receiving home care, home care clients and nursing home residents.

**Results:**

The mean HUI3 and interRAI HRQoL index global scores declined from independent healthy older persons to home care clients, followed by nursing home residents. For the home care and nursing home populations, the interRAI HRQoL global estimates tended to be lower than HUI3 global scores obtained from survey respondents. While there were some statistically significant age, sex and diagnostic group differences in global scores and within attributes, the most notable differences were between populations from different care settings.

**Discussion:**

The present study provides strong evidence for the validity of the interRAI HRQoL based on comparisons of distributional properties with those obtained with survey data based on the HUI3. The results demonstrate the importance of admission criteria for home care and nursing home settings, where function plays a more important role than demographic or diagnostic criteria. The interRAI HRQoL has a distinct advantage because it is gathered as part of normal clinical practice in care settings where interRAI instruments are mandatory and are used to assess all eligible persons in those sectors. In particular, those with severe cognitive and functional impairments (who tend to be under-represented in survey data) will be evaluated using the interRAI tools. Future research should build on this work by providing direct, person-level comparisons of interRAI HRQoL index and HUI3 scores, as well as longitudinal analyses to examine responsiveness to change.

## Introduction

Although reduction of avoidable mortality has been a public health goal in most societies, there has been a growing recognition that prevention or alleviation of disability should also be a priority for health policy and service delivery [38]. The extension of life expectancy alone is generally considered less than optimal if it is not also accompanied by additional years of healthy life. Age-related diseases, as well as chronic conditions that strike earlier in the life course, including many neurological conditions, often result in prolonged periods of disability and increased health expenditures [1]. The broader outcome of disability-free life expectancy has, therefore, become a major focus for international research on the burden of disease [41].

One approach to combining information about disability with life expectancy is represented by health-adjusted life expectancy (HALE), which uses age- and sex-specific Health Utilities Index (HUI [12]) scores (obtained from population self-reported survey data) to estimate disability and conventional life tables to estimate life expectancy [47]. HALE is widely used by Statistics Canada for a variety of applications, including microsimulation models of health over the life course (e.g., [37, 48]).

However, other research in health evaluation, pharmacoeconomics, clinical trials and cost analysis employs the HUI as the primary outcome of interest without necessarily linking it to mortality data (e.g., [28]). HUI is a multi-attribute preference-weighted measure of health-related quality of life (HRQoL) that combines measures of disability, sensory performance, emotion, pain, cognition and communication into a single score ranging from 1.0 for perfect health to 0 representing dead, to a low of − 0.03 or − 0.36 for a highly impaired state considered “worse than dead”, in the HUI2 and HUI3, respectively [12, 18]. The two variants of this measure currently in use that differ in the item sets used to create the index, but they employ comparable approaches to obtain overall summary scores. The HUI 2 is based on six attributes using indicators of sensation (using a single indicator comprised of vision, hearing, speech), mobility, emotion, cognition, self-care (using a single indicator comprised of bathing, dressing, toilet use and eating), pain (focused on pain frequency, impact on activities and medication use), and fertility. The HUI 3is comprised of eight attributes including separate indicators of vision, hearing, speech, ambulation, dexterity, emotion, cognition and pain (focused on severity and impact on activities).

Horsman et al. [29] describe HUI2 and HUI3 as complementary systems, but indicate that most analyses should be based on HUI3. HUI3 is used extensively by Statistics Canada, Health Canada and the Public Health Agency of Canada in national population surveys including the longitudinal National Population Health Survey (NPHS [48]) and the Canadian Community Health Survey (CCHS [27]). Although these population surveys provide helpful information about the general population, they face a number of methodological challenges with respect to estimates in home care and nursing home settings (e.g., refusal or inability to participate due to health concerns, item non-response due to impaired cognition, exclusion from the target population [36, 50]).

An alternative source of HRQoL information for more frail or impaired populations is the interRAI family of assessment instruments, which have been adopted in nine Canadian provinces/territories for home care and nursing home settings [20, 23]. interRAI assessments include measures related to HRQoL such as functional and cognitive impairment, health status, depressive symptoms, pain vision and hearing [5, 8, 19]. The assessments are completed by trained health professionals who use all sources of information including, but not limited to, self-report. They are completed as part of normal clinical practice and, in most provinces, cover all eligible persons in the care settings for which they are mandated. Their use is supported nationally by reporting systems managed by the Canadian Institute for Health Information (CIHI). In 2014, Canada Health Infoway designated this set of assessments as meeting the criteria for the Canadian Approved Standard for the electronic medical record.

interRAI assessments can be used to derive a variety of clinical scales that can be employed in cross-sector comparisons, including measures of depressive symptoms [6, 43], pain [14], functional status [35], frailty and health instability [2, 21, 25], and cognition [34]. Wodchis et al. [45] developed a crosswalk from two interRAI assessments (home care and nursing home) to the HUI2, known as the MDS-HSI. This scale was shown to provide comparable population-level distributions as obtained with direct HUI2 measures [46] and it has been used in studies related to frail seniors in the community [49], as well as studies in nursing homes related to pressure ulcers [42, 44], pneumonia [32], *c difficile* [13], and anemia [4]. Lam and Wodchis [30] used the MDS-HSI to examine the relationship of the scale with 60 different diagnoses and 15 health conditions in nursing homes. Although the HUI2 was originally created to measure HRQoL in different settings, including nursing homes [29], it is not used in government-supported surveys of the general population in Canada, where the HUI3 is the preferred measure.

A crosswalk methodology was developed to obtain a HRQoL index comparable to HUI3 from interRAI’s assessments. The study used two of the older interRAI instruments [the Resident Assessment 2.0 (RAI 2.0) for nursing homes and the RAI-Home Care (RAI-HC)]; however, the crosswalk can also be used with the newer suite of interRAI instruments covering the full continuum of care [17]. The interRAI HRQoL index was subsequently used in a microsimulation model of the impact of neurological conditions over the life course developed by Statistics Canada as part of the National Population Health Study of Neurological Conditions (NPHSNC) [9]. The present paper describes the development and validity of the methodology.

## Methods

### Study samples

The study samples for interRAI-related analyses were drawn from three datasets: (i) a small sample of well-elderly individuals in Newfoundland assessed with the RAI-HC (*n* = 346) as part of a 2001 community intervention study [11]; (ii) Ontario long-stay home care clients (expected to receive services for 60 days or more) assessed with the RAI-HC in 2012 (*n* = 256,348); and (iii) Ontario nursing home residents assessed with the RAI 2.0 in 2012 (*n* = 308,343). The home care and nursing home samples effectively represent census-level data, because the mandated use of interRAI instruments applies to all persons receiving care in those settings. However, the well-elderly sample was a random sample of persons aged 70 years and older from the community that excluded individuals receiving any home care or community support services.

The newest versions of the interRAI assessments include about 320 individual items in both the nursing home and home care versions [8, 17]. These assessments are usually done by trained health professionals who use all sources of information to complete standardized items dealing with domains like cognition, mood, behavior, functional status, health problems, skin conditions, nutritional status, psychosocial well-being, environmental factors, service use, medical procedures and caregiving arrangements. The reliability and validity of these instruments have been studied extensively (see, for example, [15, 16, 22, 24, 26, 31, 40]). In addition to the individual items, interRAI assessments include numerous embedded summary scales related to clinical issues like cognition [34], functional status [35], depressive symptoms [43], pain [14], aggressive behavior [39], and medical instability [25]. The ranges of items and scales vary, but in all cases higher scores correspond to higher levels of impairment.

Table 1 provides an overview of the demographic and clinical characteristics of the three interRAI study samples. For all three samples, two-thirds were female and the majority were 75 years of age or older. Nursing homes had the largest proportion of persons aged 85 years and older, with about half the sample in that age group compared to about one-third in home care and 17% of the well-elderly sample. Of the diagnoses considered, the lowest prevalence rates were in the well-elderly sample. Rates were comparable in the home care and nursing home samples, except psychiatric diagnoses which were substantially higher among nursing home residents. There were pronounced differences between these samples when various interRAI scale distributions were compared. For example, concerning Cognitive Performance Scale (CPS) scores, almost the entire well-elderly sample was cognitively intact (i.e., CPS = 0), whereas more than 50% of nursing home residents were moderately impaired or more (CPS > 3). Similarly, those in the well-elderly sample were almost all independent in function according to the Activities of Daily Living (ADL) hierarchy scale (i.e., ADL = 0), but two-thirds of nursing home residents had moderately impaired physical function or worse (ADL ≥ 3). Pain scale scores were similarly distributed in the three study samples, whereas the Changes in Health, End-stage disease, Signs and Symptoms (CHESS) scale score indicated the highest rates of frailty or health instability in the home care and nursing home samples. Depressive symptoms were largely absent in the well-elderly sample, but scores of 3 or more on the Depression Rating Scale (DRS) were evident for 17% of home care clients and one quarter of nursing home residents.

**Table 1:** Percentage distributions of demographic, diagnostic, and clinical indicators among persons assessed with interRAI instruments, by setting/subsample

	Well-elderly sample, Newfoundland, 2001	Long-stay home care clients, Ontario, 2012	Nursing home residents, Ontario, 2012
Sample size	345	308,356	256,348
Female (%)	67.5	63.2	66.8
Age group (%)			
0–44	–	3.5	0.7
45–54	–	5.1	1.5
55–64	–	9.6	4.0
65–74	2.0	15.7	10.0
75–84	80.6	34.3	33.8
85+	17.4	31.7	50.0
Diagnosis			
Heart failure (%)	3.5	10.5	13.2
Emphysema/COPD (%)	2.6	16.3	12.8
Diabetes (%)	16.5	25.7	23.3
Cancer (%)	4.3	16.4	10.5
Any psychiatric condition (%)	S	14.2	27.8
Cognitive Performance Scale (CPS) score (%)			
0	97.4	44.1	16.4
1—2	2.0	45.4	32.0
3–4	S	7.1	34.0
5–6	S	3.4	17.7
Activities of Daily Living Hierarchy (ADL) score (%)			
0	99.1	63.7	9.0
1–2	S	24.3	26.6
+	S	12.0	64.4
Pain scale (%)			
0	71.1	33.1	53.9
1–2	24.6	53.1	42.8
3+	4.3	13.8	3.3
CHESS scale (%)			
0	87.0	27.9	52.0
1–2	12.7	57.2	42.1
3+	S	14.8	5.9
Depression Rating Scale (DRS) (%)			
0	96.2	59.1	44.2
1–2	3.2	23.7	30.5
3+	S	17.2	25.4

“S” designates cells suppressed due to *n* < 10

Comparative data from national surveys were used to estimate the distributions of HUI3 in samples where it was included in the survey questionnaire. In this case, cycle 4.2 (2009) of the CCHS (also called CCHS-Healthy Aging) was used to provide data on HUI3 in the adult (age 45 years and older) general household population by age and sex (*n* = 32,005), including a subset of individuals who self-reported receipt of formal (paid) home care services (*n* = 3083). The subgroups of the CCHS sample were used to make comparisons with the interRAI samples of the well-elderly and home care clients, depending on whether they reported receiving formal home care services or not. The interRAI nursing home sample was compared with data from the longitudinal NPHS, which was first conducted in 1994 with 2-year follow-ups until 2010. The NPHS included individuals living in “facilities for the aged”, and HUI3 data were also available for that subsample (*n* = 842) (Data not shown).

### Development of interRAI HRQoL index

The interRAI HRQoL was designed to replicate the theoretical and clinical logic used to construct the HUI 3 as a measure of health-related quality of life. It was not the intent to create a fully new measure, but rather to develop a means of obtaining scores comparable to the HUI 3 from interRAI assessments. The initial work on the crosswalk was done by individual members of an expert panel comprised of two clinicians (nurse and social worker) and six researchers (mix of backgrounds in health service research, statistics, epidemiology) familiar with interRAI assessments and HUI3. Each individual independently selected items or scales from interRAI assessments that were conceptually similar to the measures included in the HUI3. Next, each member proposed cut-off values in the interRAI items and scales to match domain-specific severity levels in the HUI3. The expert panel then met in-person as a group to discuss points of discrepancy in their individual recommendations to achieve a consensus on which items, scales and cut-points would be used to match the HUI3 classification scheme. The expert panel considered clinical issues for determining individual domains or cut-points, but they also examined the distributions of the HUI3 attributes from survey data compared with the distributions obtained from the interRAI crosswalk as a guide for approximate proportions to expect at different levels of severity of impairment in different care settings. Only one in-person meeting of the panel was required to come to a consensus on the coding rules; however, some additional analyses were done after that meeting to make minor refinements to coding rules. Those issues were resolved through telephone follow-ups to review the needed adjustments.

A second stage of scrutiny was an independent review provided by a committee of interRAI Fellows from eight countries (Canada, United States, Finland, France, Poland, Czech Republic, Belgium, Australia) that included clinicians with expertise in geriatric medicine (5), social work (2), rehabilitation (1) as well as four health services researchers. The committee reviewed the detailed coding instructions for the crosswalk and the specific items, scales and cut-points that were used. Based on that review, the committee endorsed use of the HRQoL as a measure of health-related quality of life based on interRAI systems.

### RAI 2.0 (long-term care homes) crosswalk

Table 2 shows how RAI 2.0 items and scales were mapped to the HUI3 attribute levels. The vision attribute is measured by a combination of the RAI 2.0 vision and visual appliances items. The hearing attribute is measured by the hearing item and either of two hearing aid items (present and used regularly or present and not used regularly). The speech attribute is measured by the RAI 2.0 making one’s self understood item. The ambulation attribute is measured by an item regarding locomotion on the nursing unit and other items regarding the modes of locomotion (cane, walker or crutch and 3 wheelchair-related items: wheeled self, other person wheeled, wheelchair primary mode of locomotion). The dexterity attribute did not have a directly corresponding RAI 2.0 item. Therefore, the crosswalk used the ADL eating item because the dexterity attribute of the HUI3 emphasized hand and arm use. In measuring dexterity, the CPS [34] was used to distinguish those whose eating performance was impaired due to cognitive impairment rather than physical disability. Whereas the HUI3 emotion attribute focuses on happiness, the RAI 2.0 does not include a direct measure of happiness. Therefore, as was done with the MDS-HSI, the emotion attribute was operationalized using the DRS [6]. The RAI 2.0 items for cognitive skills for decision-making and short-term memory items were used for the cognition attribute. The interRAI Pain scale [14], which combines pain frequency and intensity items, was used for the pain attribute.

**Table 2:** RAI 2.0 scale items and levels used to develop HUI3 crosswalk, with associated HUI3 attribute levels and assigned utility scores

HUI3 attribute	RAI 2.0 items or scales	RAI 2.0 scale level	HUI3 attribute level	Assigned utility weight
Vision	D1—vision (with glasses if used) D3—use of visual appliances (e.g., glasses)	Adequate vision without use of visual appliances	1	1.00
		Adequate vision with the use of visual appliances	2 or 3	0.974
		Impaired vision	4	0.84
		Moderately impaired vision	5	0.75
		Highly or severely impaired vision	6	0.61
Hearing	C1—hearing (with appliance if used) C2a and C2b—hearing aid use	Adequate hearing, without the use of a hearing aid	1	1.00
		Adequate hearing, with the use of a hearing aid	2	0.95
		Minimal difficulty in hearing, without the use of a hearing aid	2	0.95
		Minimal difficulty in hearing, with the use of a hearing aid	3	0.89
		Hears in special situations only	4 or 5	0.789
		Highly impaired hearing	6	0.61
Speech	C4—making one’s self understood	Understood when expressing information	1	1.00
		Usually understood	2 or 3	0.909
		Sometimes understood	4	0.81
		Rarely or never understood	5	0.68
Ambulation	G1ea—self-performance: locomotion on the nursing unit G5a through d—modes of locomotion (mobility aid use)	Independent locomotion, without the use of walking aid	1 or 2	0.997
		Independent locomotion, with the use of a walking aid	3	0.86
		Supervision in locomotion, without use of walking aid	3	0.86
		Supervision in locomotion, with use of walking aid	4	0.73
		Limited assistance in locomotion	4	0.73
		Extensive assistance in locomotion	5	0.65
		Any wheelchair use	5	0.65
		Total dependence, or locomotion does not occur	6	0.58
Dexterity	G1ha—self-performance: eating Cognitive Performance Scale (CPS)	Independent eating	1	1.00
		Requires supervision only for eating	1	1.00
		If individual has moderate or greater cognitive impairment (CPS ≥ 3)		
		Limited assistance in eating	2 or 3	0.919
		Extensive assistance in eating	2 or 3	0.919
		Total dependence in eating	2 or 3	0.919
		If individual has mild cognitive impairment (CPS < 3)		
		Limited assistance in eating	4	0.76
		Extensive assistance in eating	5	0.65
		Total dependence in eating	6	0.56
		Eating did not occur	6	0.56
Emotion	Depression Rating Scale (DRS)	DRS = 0	1	1.00
		DRS = 1	2	0.95
		DRS = 2	3	0.85
		DRS = 3 or 4	4	0.64
		DRS = 5 to 14	5	0.46
Cognition	B2a—short term memory B4—cognitive skills for decision making	Independent decision making A Intact short term memory	1	1.00
		Modified independence in decision making AND Intact short term memory	2	0.92
		Independent decision making AND Short term memory problems	3	0.95
		Modified independence in decision making AND Short term memory problems	4	0.83
		Moderately impaired decision making	5	0.60
		Severely impaired decision making AND Intact short term memory	5	0.60
		Severely impaired decision making AND Short term memory problems	6	0.42
Pain	Pain scale	Pain scale = 0	1	1.00
		Pain scale = 1	2	0.96
		Pain scale = 2	3	0.90
		Pain scale = 3	4 or 5	0.643

In certain cases, response options available from RAI 2.0 did not correspond to a single HUI3 attribute level. For example, regarding the vision attribute, it was not possible to differentiate someone whose use of eyeglasses improves both near- and short-sightedness (HUI3 vision level 2) and those whose use of eyeglasses does not allow them to see at a distance (HUI3 vision level 3) using the available RAI 2.0 items.

### RAI-HC (home care) crosswalk

The HUI3 crosswalk for the RAI-HC followed the same basic logic as was used with the RAI 2.0 with a limited number of exceptions. First, because vision and hearing appliances could not be used to modify those two domains, the weighted mean scores of the multi-attribute weights of being able to see or hear with or without those appliances was assigned based on the number of persons with each of those characteristics in the available survey data. Second, making one’s self understood, locomotion and eating had an additional response level in the RAI-HC compared with RAI 2.0, but these were collapsed to match the RAI 2.0 crosswalk code (see Table 3).

**Table 3:** RAI-HC scale items and levels used to develop HUI3 crosswalk, with associated HUI3 attribute levels and assigned utility scores

HUI3 attribute	RAI-HC items or scales	RAI-HC scale level	HUI3 attribute level	Assigned utility weight
Vision	D1—vision (with glasses if used)	Adequate vision	1, 2 or 3	0.989
		Impaired vision	4	0.84
		Moderately impaired vision	5	0.75
		Highly or severely impaired vision	6	0.61
Hearing	C1—hearing (with appliance if used)	Adequate hearing	1	1.00
		Minimal difficulty in hearing	2 or 3	0.934
		Hears in special situations only	4 or 5	0.791
		Highly impaired hearing	6	0.61
Speech	C2—making self understood	Understood when expressing information	1	1.00
		Usually understood	2	0.94
		Often understood	3	0.89
		Sometimes understood	4	0.81
		Rarely or never understood	5	0.68
Ambulation	H2c—locomotion in home H4a—primary modes of locomotion (mobility aid use)	Independent locomotion, without the use of walking aid	1 or 2	0.999
		Independent locomotion, with the use of a walking aid	3	0.86
		Setup, supervision or limited assistance in locomotion, without use of walking aid	3	0.86
		Independent, setup or supervision in locomotion, with use of scooter	4	0.73
		Setup, supervision or limited assistance in locomotion with use of a walking aid	4	0.73
		Independent, setup or supervision in locomotion with wheelchair use	5	0.65
		Limited, extensive or maximal assistance in locomotion with scooter or wheelchair use	5	0.65
		Extensive or maximal assistance in locomotion and no scooter or wheelchair use	5	0.65
		Total dependence in locomotion, or activity did not occur	6	0.58
Dexterity	H2g—self-performance: eating Cognitive Performance Scale (CPS)	Independent eating	1	1.00
		Requires setup or supervision only for eating	1	1.00
		If individual has moderate or greater cognitive impairment (CPS ≥ 3)		
		Limited assistance in eating	2 or 3	0.945
		Extensive or maximal assistance in eating	2 or 3	0.945
		Total dependence in eating	2 or 3	0.945
		If individual has mild cognitive impairment (CPS < 3)		
		Limited assistance in eating	4	0.76
		Extensive assistance in eating	5	0.65
		Total dependence in eating	6	0.56
		Eating did not occur	6	0.56
Emotion	Depression Rating Scale (DRS)	DRS = 0	1	1.00
		DRS = 1	2	0.95
		DRS = 2	3	0.85
		DRS = 3 or 4	4	0.64
		DRS = 5 to 14	5	0.46
Cognition	B2a—short term memory B4—cognitive skills for decision making	Independent decision making and intact short term memory	1	1.00
		Modified independence in decision making AND Intact short term memory	2	0.92
		Independent decision making AND Short term memory problems	3	0.95
		Minimally or moderately impaired decision making AND Intact short term memory	3	0.95
		Modified independence in decision making AND Short term memory problems	4	0.83
		Minimally or moderately impaired decision making AND Short term memory problems	5	0.60
		Severely impaired decision making AND Intact short term memory	5	0.60
		Severely impaired decision making AND Short term memory problems	6	0.42
Pain	Pain scale	Pain scale = 0	1	1.00
		Pain scale = 1	2	0.96
		Pain scale = 2	3	0.90
		Pain scale = 3	4 or 5	0.678

With respect to the new suite of interRAI instruments [17], the coding rules for the RAI-HC would be most appropriate given that they correspond more closely with the new versions of item codes in those instruments.

### Calculation of the interRAI HRQoL index score

The HUI3 has eight attributes of HRQoL: vision, hearing, speech, cognition, mobility, dexterity, emotion, pain. The interRAI assessments have items and scales that correspond with most of the HUI3 attributes, but there are some differences in the available items in the nursing home and home care instruments due to their different developmental timelines.

For interRAI items that were mapped to a single HUI3 attribute level, the original utility weights were retained [12]. In cases where interRAI items mapped to more than one HUI3 attribute level, a weighted average utility score was estimated. The weights were based on the distribution of the underlying HUI3 attribute levels in the relevant survey population (i.e., CCHS-Healthy Aging for the home care sample, and NPHS institutional for the nursing home sample). Utility weights assigned to interRAI items are given in Table 2 for the home care population, and in Table 3 for the nursing home population. The standard HUI3 formula [12] was applied using the product of utility weight by domain as follows:

$${\text{Global HRQoL Score}}={\text{1}}.{\text{371}}\left( {{{\text{u}}_{{\text{vision}}}} \times {{\text{u}}_{{\text{hearing}}}} \times {{\text{u}}_{{\text{speech}}}} \times {{\text{u}}_{{\text{ambulation}}}} \times {{\text{u}}_{{\text{dexterity}}}} \times {{\text{u}}_{{\text{emotion}}}} \times {{\text{u}}_{{\text{cognition}}}} \times {{\text{u}}_{{\text{pain}}}}} \right) - 0.{\text{37}}$$

### Analysis

Once consensus was reached for the coding rules to create the interRAI HRQoL index, the distributions of the global scores and attributes were compared against distributions of the HUI3 obtained in similar survey populations. Mean scores and percentages were compared in home care and nursing homes between the interRAI groups and their corresponding comparison samples from the CCHS and NPHS. Where confidence intervals from the survey estimates include the interRAI mean or percentage, it can be assumed that the differences between means are not statistically significant. For the sample of well-elderly persons assessed with the RAI-HC and the CCHS general population sample, a test of comparison of means was performed.

## Results

Several criteria were used to examine the performance of the interRAI HRQoL compared with the HUI 3. First, scores obtained from surveys of corresponding clinical (i.e., well-elderly, home care, nursing homes) populations should be comparable to those obtained from interRAI assessments. Second, settings serving persons with heavier care needs should have lower mean global scores as well as higher rates of impairment in the specific attributes. Third, the HRQoL global score should be associated with factors likely to be influenced by the severity of impairments in health-related quality of life.

Table 4 provides the mean global HUI3 scores for CCHS respondents aged 65 and older who were not recipients of home care services compared with the interRAI HRQoL in the well-elderly sample who were also not receiving home care or community support services. For the CCHS sample, HUI3 scores tended to decline among older age groups, but there were no pronounced differences between males and females. For the well-elderly sample, almost all hypotheses of equality of means between HUI and interRAI HRQoL by age and gender were not rejected. Where the hypothesis of equality of means was rejected, the mean interRAI HRQoL scores were higher in the well-elderly sample (4 groups).

**Table 4:** Global HUI3 values for CCHS survey respondents with no homecare (formal or informal) compared with well-elderly study participants with no home care or community support services

Sex	Age group	Well- elderly intervention study with no home care, Newfoundland (*n* = 346)	Respondents to the Canadian Community Health Survey, Cycle 4.2 (Healthy Aging) receiving no formal home care services, Canada, 2009	
		Mean interRAI HRQoL (95%CL)	*n*	Mean HUI 3 global score (95% CL)
Female	65–74	S	2942	0.86 (0.86, 0.87)
	75–84	0.88 (0.86, 0.91)	1947	0.84 (0.83, 0.85)
	85+	**0.86 (0.81, 0.92)**	935	**0.76 (0.74, 0.79)**
Male	65–74	S	2803	0.87 (0.86, 0.88)
	75–84	0.88 (0.85, 0.92)	1478	0.84 (0.83, 0.85)
	85+	**0.90 (0.82, 0.98)**	666	**0.73 (0.69, 0.77)**
Both sexes	65–74	0.68 (0.44, 0.93)	5745	0.87 (0.86, 0.87)
	75–84	**0.88 (0.86, 0.90)**	3425	**0.84 (0.83, 0.85)**
	85+	**0.87 (0.82, 0.91)**	1601	**0.75 (0.73, 0.77)**

Bolded values indicate cells where hypothesis of equality of means in the two groups was rejected

“S” designates cells suppressed due to *n* < 10

Table 5 provides the mean global HUI3 scores and the proportion with moderate or severe limitations based on previously reported cut-point [33] in each attribute for long-stay home care clients and CCHS respondents who reported that they received government-funded or private-pay home care. Among long-stay home care clients, those aged 85 years and older had lower global interRAI HRQoL scores compared to younger home care clients. However, across age groups, where the hypothesis of equality of means was rejected the home care sample had lower mean global scores compared with the well-elderly sample (see Table 4). Whereas the well-elderly sample had global scores between 0.68 and 0.90 for both sexes, home care clients’ scores ranged between 0.34 and 0.45 for both sexes (see also Table 5). According to Horsman et al. [29] differences of 0.03 in global HUI3 scores are considered clinically significant; however, Drummond [10] suggests that 0.01 may also be important. Mean global HUI3 scores were generally higher among CCHS respondents who reported receiving formal home care services compared to interRAI HRQoL scores among long-stay home care clients (Table 5). There were relatively few differences in the interRAI and survey estimates across age or sex groups in the proportion of long-stay home care clients with moderate or severe limitations for the hearing, speech and dexterity attributes. However, there are pronounced differences among the ambulation, pain, cognition and vision attributes, with interRAI-assessed clients tending to show higher rates of limitation compared to CCHS respondents receiving home care services.

**Table 5:** Mean global HUI3 score and proportion in moderate or severe disability, by HUI3 attribute, by age group and sex, long-stay home care clients in Ontario (2012) and Canadian Community Health Survey Cycle 4.2 respondents who self-reported receiving formal home care services (2009)

	Long-stay home care clients, Ontario, 2012^a^				Respondents to the Canadian Community Health Survey, Cycle 4.2 (Healthy Aging) receiving formal home care services, Canada, 2009			
	< 65	65–74	75–84	85+	45–64	65–74	75–84	85+
Sample size								
Male	24,252	20,401	39,250	29,645	163	167	219	434
Female	31,964	28,094	66,565	68,172	275	276	640	1009
Total	56,216	48,495	105,815	97,817	438	443	759	1443
Mean global HUI3 score								
Male	0.45	0.45	0.40	0.34	**0.54 (0.48, 0.60)**	**0.59 (0.54, 0.65)**	**0.52 (0.47, 0.57)**	**0.51 (0.46, 0.55)**
Female	0.43	0.44	0.40	0.34	**0.55 (0.48, 0.62)**	**0.53 (0.48, 0.57)**	**0.54 (0.50, 0.57)**	**0.45 (0.41, 0.48)**
Total	0.44	0.44	0.40	0.34	**0.55 (0.50, 0.60)**	**0.55 (0.52, 0.59)**	**0.53 (0.50, 0.56)**	**0.46 (0.43, 0.49)**
% Vision* attribute ≥ 4								
Male	18.5	20.5	24.6	32.5	*n* < 10	*n* < 10	**8.0** ^**E**^ **(4.6, 13.6)**	**11.5** ^**E**^ **(7.1, 18.0)**
Female	18.3	20.6	25.1	35.2	F	F	**6.4** ^**E**^ **(4.2, 9.8)**	**13.2 (10.0, 17.2)**
Total	18.4	20.5	24.9	34.4	**4.6** ^**E**^ **(2.4, 8.5)**	**3.7** ^**E**^ **(2.2, 6.3)**	**6.9** ^**E**^ **(4.9, 9.6)**	**12.7 (9.9, 16.1)**
% Hearing attribute ≥ 4								
Male	2.9	6.0	14.3	30.0	F	F	**23.4** ^**E**^ **(15.5, 33.7)**	31.5 (23.8, 40.5)
Female	2.1	3.9	9.0	23.3	F	5.1^E^ (2.8, 9.0)	8.1^E^ (5.2, 12.4)	24.5 (19.9, 29.8)
Total	2.5	4.8	11.0	25.3	F	5.4^E^ (3.4, 8.5)	12.8 (9.4, 17.2)	26.5 (22.1, 31.3)
% Speech* attribute ≥ 4								
Male	5.8	3.9	4.0	3.6	S	S	S	S
Female	4.2	2.6	2.9	3.5	S	S	S	S
Total	4.9	3.2	3.3	3.5	S	S	S	F
% Ambulation attribute ≥ 3								
Male	48.6	52.8	59.1	70.0	**16.1** ^**E**^ **(10.3, 24.4)**	**22.0** ^**E**^ **(15.4, 30.3)**	**40.0 (31.4, 49.4)**	**45.6 (38.4, 53.0)**
Female	48.9	55.3	61.8	73.2	**24.4** ^**E**^ **(17.0, 33.8)**	**34.8 (27.7, 42.7)**	**46.3 (40.6, 52.1)**	**62.0 (56.4, 67.3)**
Total	48.8	54.3	60.8	72.2	**21.1 (15.8, 27.7)**	**29.6 (24.4, 35.4)**	**44.4 (39.6, 49.2)**	**57.4 (52.8, 61.8)**
% Dexterity* attribute ≥ 4								
Male	5.1	3.7	2.6	2.5	S	S	S	S
Female	3.4	2.3	1.8	2.1	S	S	3.0^E^ (1.6, 5.5)	3.1^E^ (1.9, 5.2)
Total	4.1	2.9	2.1	2.2	F	2.4^E^ (1.0, 4.2)	2.5^E^ (1.4, 4.3)	2.5^E^ (1.6, 4.0)
%Emotion attribute ≥ 3								
Male	28.9	27.9	25.8	21.8	28.1^E^ (15.9, 44.6)	**14.9** ^**E**^ **(8.4, 25.0)**	**13.5** ^**E**^ **(8.2, 21.5)**	**7.1** ^**E**^ **(4.4, 11.1)**
Female	38.7	34.6	29.2	23.2	**13.1** ^**E**^ **(7.4, 22.1)**	**12.1** ^**E**^ **(8.0, 17.8)**	**12.2 (9.0, 16.4)**	**7.9** ^**E**^ **(5.5, 11.3)**
Total	34.5	31.8	27.9	22.8	**19.0** ^**E**^ **(12.5, 27.7)**	**13.2** ^**E**^ **(9.4, 18.3)**	**12.6 (9.8, 16.1)**	**7.7 (5.8, 10.2)**
% Cognition attribute ≥ 4								
Male	22.4	29.9	41.3	46.4	19.9^E^ (12.3, 30.6)	**15.7** ^**E**^ **(9.7, 24.4)**	**25.8 (18.9, 34.2)**	**26.5 (20.9, 33.1)**
Female	17.9	23.9	36.1	43.9	16.4^E^ (11.2, 23.3)	**9.6** ^**E**^ **(6.1, 14.6)**	**17.2 (13.1, 22.4)**	**28.2 (22.8, 34.2)**
Total	19.8	26.4	38.0	44.7	17.8 (13.4, 23.2)	**12.0** ^**E**^ **(8.6, 16.6)**	**19.9 (16.2, 24.2)**	**27.7 (23.5, 32.3)**
% Pain attribute ≥ 3								
Male	54.8	49.8	46.0	43.6	40.2^E^ (28.0, 53.8)	39.9 (29.2, 51.8)	**34.0 (25.8, 43.4)**	**32.5 (26.0, 39.8)**
Female	63.6	62.5	59.6	54.7	**50.5 (39.5, 61.5)**	56.9 (49.3, 64.2)	**43.1 (37.5, 48.8)**	**38.3 (33.1, 43.9)**
Total	59.8	57.1	54.5	51.4	**46.5 (37.9, 55.3)**	**50.0 (43.4, 56.6)**	**40.3 (35.6, 45.2)**	**36.7 (32.5, 41.2)**

Bolded values indicate cells where confidence limits for CCHS estimates do not contain interRAI values

“E” designates need to use with caution (coefficient of variation between 16.6 and 33.3%)

“F” designates NPHS cells suppressed due to high coefficient of variation (33.3% or greater)

“S” designates cells suppressed due to *n* < 10

*In general, HUI3 attributes at level 3 or greater (level 4 or greater for cognition) are considered “moderate or severe” disability. However, because interRAI items could not always distinguish attribute levels 2 and 3, on certain scales these levels were assigned weighted averages based on distributions reported in CCHS. Therefore, for the asterisked scales, “moderate or severe” disability categories were changed to be level 4 or above

^a^Ontario home care values do not include 95% confidence limits because they represent census values for that population, whereas estimates from the CCHS include the estimated percentages and 95% confidence limits for those estimates

Table 6 provides the mean global interRAI HRQoL scores and the proportion with moderate or severe impairment within each attribute for residents of nursing home facilities assessed with the RAI 2.0. Similar estimates based on HUI3 are given for NPHS respondents residing in facilities for the aged between 1994 and 2010. For both samples, there were substantially lower global scores in nursing home residents in all age and sex groups compared with the community samples shown in Tables 4 and 5. The average interRAI HRQoL scores were generally lower than the corresponding HUI3 scores from the NPHS (scores ranged between 0.09 and 0.20 compared with 0.15 and 0.38, respectively). Unlike what was observed with the community samples, interRAI HRQoL scores decreased with increased age among nursing home residents; however, mean HUI3 scores did not differ substantially by age in the NPHS. The sex differences for global scores were small (females had lower scores) for the interRAI HRQoL, because of large uncertainty in HUI3 for males: mean HUI3 scores were not compared between sexes.

**Table 6:** Mean global HUI3 and percentage with moderate or severe disability, by HUI3 attribute, by age group and sex

	Ontario nursing home residents (*n* = 256,348)				National Population Health Survey respondents (1994 through 2010) living in “facilities for the aged”^a^			
	00–64	65–74	75–84	85+	45–64	65–74	75–84	85+
Sample size								
Male	7827	11,809	32,114	33,424	47	103	205	179
Female	7991	13,922	54,564	94,697	53	156	463	762
Total	15,818	25,731	86,678	128,121	100	259	668	941
Mean global HUI3 score								
Male	0.20	0.18	0.15	0.13	**0.38** ^**E**^ **(0.23, 0.53)**	F	F	F
Female	0.15	0.16	0.13	0.09	F	**0.28** ^**E**^ **(0.19, 0.38)**	**0.25** ^**E**^ **(0.16, 0.33)**	**0.19 (0.14, 0.24)**
Total	0.17	0.17	0.14	0.10	F	0.23^E^ (0.14, 0.32)	0.19^E^ (0.11, 0.26)	**0.15 (0.11, 0.20)**
Vision*, attribute ≥ 4								
Male	30.9	34.9	39.9	47.3	F	S	**20.6** ^**E**^ **(11.0, 35.1)**	38.7^E^ (22.2, 58.2)
Female	30.5	34.1	39.6	49.8	F	F	**21.3** ^**E**^ **(13.8, 31.4)**	**26.5 (20.8, 33.1)**
Total	30.7	34.5	39.7	49.2	F	**12.1** ^**E**^ **(7.1, 20.0)**	**21.0** ^**E**^ **(14.9, 28.9)**	**29.7 (23.4, 36.9)**
Hearing, attribute ≥ 4								
Male	3.9	6.0	11.9	24.2	S	S	F	16.9 (8.8, 29.9)
Female	3.5	4.6	8.3	19.7	S	F	**13.3 (8.4, 20.6)**	20.0 (15.7, 25.2)
Total	3.7	5.2	9.7	20.9	S	7.8 (4.4, 13.7)	**15.9 (10.0, 24.3)**	19.2 (15.3, 23.8)
Speech*, attribute ≥ 4								
Male	19.7	19.9	19.2	14.3	17.1^E^ (8.8, 30.7)	F	F	F
Female	19.5	19.2	18.7	15.9	*n* < 10	F	14.2^E^ (8.5, 23.0)	**9.7 (7.0, 13.3)**
Total	19.6	19.5	18.9	15.5	F	14.6^E^ (7.6, 26.3)	14.7^E^ (9.8, 21.5)	11.2^E^ (7.6, 16.2)
Ambulation, attribute ≥ 3								
Male	79.7	80.7	84.8	91.1	**40.2** ^**E**^ **(22.2, 61.2)**	67.8 (47.7, 83.0)	**61.5 (41.6, 78.2)**	**75.3 (55.7, 88.1)**
Female	84.0	81.0	85.9	93.7	73.1 (48.9, 88.5)	**49.8** ^**E**^ **(34.0, 65.5)**	**62.1 (51.3, 71.8)**	**69.5 (61.2, 76.7)**
Total	81.9	80.9	85.5	93.0	**60.6 (42.1, 76.4)**	**56.2 (43.1, 68.5)**	**61.9 (52.4, 70.6)**	**71.0 (63.2, 77.6)**
Dexterity*, attribute ≥ 4								
Male	9.5	6.0	5.0	5.0	*n* < 10	F	**30.5** ^**E**^ **(16.1, 50.1)**	F
Female	9.8	6.2	4.6	4.7	F	F	**16.8** ^**E**^ **(10.0, 26.8)**	**14.3** ^**E**^ **(10.1, 19.8)**
Total	9.7	6.1	4.8	4.7	F	**13.2** ^**E**^ **(6.8, 24.0)**	**21.2** ^**E**^ **(14.3, 30.3)**	**14.6 (10.8, 19.5)**
Emotion, attribute ≥ 3								
Male	35.3	36.0	35.9	33.1	F	42.5^E^ (23.2, 64.5)	**63.4 (46.5, 77.6)**	60.6 (44.6, 74.5)
Female	46.3	44.0	43.4	41.3	54.5^E^ (27.7, 79.0)	**18.6** ^**E**^ **(10.9, 29.9)**	**31.7 (23.3, 41.4)**	**26.3 (20.8, 32.6)**
Total	40.9	40.3	40.6	39.2	41.3^E^ (22.9, 62.5)	**27.4** ^**E**^ **(17.7, 39.9)**	42.1 (32.6, 52.3)	35.3 (28.6, 42.7)
Cognition, attribute ≥ 4								
Male	53.9	62.2	70.1	69.9	44.2^E^ (27.1, 62.7)	66.5 (45.5, 82.5)	67.3 (49.1, 81.5)	72.0 (56.5, 83.6)
Female	52.5	59.5	68.8	71.3	55.5^E^ (27.5, 80.5)	63.8 (47.4, 77.5)	66.7 (56.2, 75.7)	**63.4 (56.1, 70.2)**
Total	53.2	60.7	69.3	70.9	51.2^E^ (31.7, 70.4)	64.7 (52.3, 75.4)	66.9 (57.3, 75.2)	65.7 (59.1, 71.7)
Pain, attribute ≥ 3								
Male	21.1	19.4	17.6	18.1	S	F	**37.3** ^**E**^ **(20.9, 57.4)**	28.1^E^ (15.0, 46.3)
Female	27.5	26.8	23.9	23.0	S	18.6^E^ (10.8, 30.3)	22.4 (16.4, 29.8)	**28.4 (22.7, 35.0)**
Total	24.3	23.4	21.6	21.7	F	21.7^E^ (13.6, 32.8)	27.4 (20.1, 36.1)	**28.3 (22.5, 35.0)**

Bolded values indicate cells where confidence limits for NPHS estimates do not overlap with interRAI values

“E” designates need to use with caution (coefficient of variation between 16.6% and 33.3%)

“F” designates NPHS cells suppressed due to high coefficient of variation (33.3% or greater)

“S” designates cells suppressed due to *n* < 10

*In general, HUI3 attributes at level 3 or greater (level 4 or greater for cognition) are considered “moderate or severe” disability. However, because interRAI items could not always distinguish attribute levels 2 and 3, on certain scales these levels were assigned weighted averages based on distributions reported in NPHS. Therefore, for the asterisked scales, “moderate or severe” disability categories were changed to be level 4 or above

^a^NPHS values include the estimated percentages and 95% confidence limits for those estimates. Ontario LTC values do not include 95% confidence limits because they represent census values for that population.

**Fig. 1:**
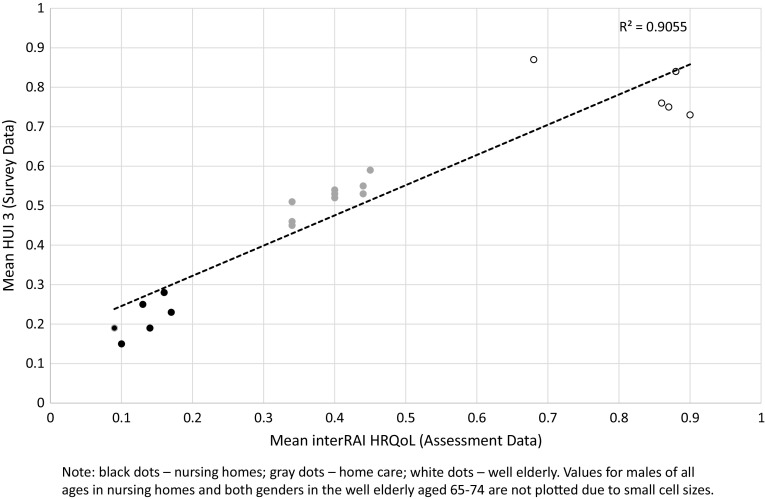
Scatter plot of mean scores for HUI 3 and interRAI HRQoL obtained from survey and assessment data, respectively, for corresponding age and gender groups by setting

To examine the degree of correspondence between interRAI HRQoL and HUI3 scores, the mean global score for each age-sex group in the three samples (well-elderly, home care, nursing homes) were compared in a scatter plot (see Fig. 1). The *R*^2^ for the 22 pairs of global scores with values in both data sources in these three settings (see Tables 4, 5, 6) was 0.91, indicating a very strong association between the two global health related quality of life scores.

Although concurrent interRAI HRQoL and HUI 3 scores are not available in this study to establish criterion validity of the HRQoL against HUI 3, the interRAI assessment data may be used to examine patterns of association as evidence of convergent validity for the interRAI HRQoL. Table 7 shows the mean interRAI HRQoL scores for home care clients and nursing home residents by end-stage disease where prognosis is less than 6 months to live. Although the mean HRQoL scores differ between the same prognosis groups in the two settings (reflecting more advanced illness and disability in nursing homes), persons with a prognosis of less than 6 months to live have worse HRQoL scores than those *within* their care setting who have a longer prognosis.

**Table 7:** Mean global HRQoL (95% CL) by end-stage disease (prognosis < 6 months) and care setting

End-stage disease	Home care			Nursing home		
	*N*	Mean HRQoL	95% CL	*N*	Mean HRQoL	95% CL
No	304,105	0.40	0.39–0.40	253,135	0.13	0.12–0.13
Yes	4341	0.31	0.30–0.32	3213	0.00	− 0.01–0.01

Table 8 collapsed HRQoL into five groups of roughly 0.25 increments in both care settings. In home care, there is a clear difference in the mean hours of informal support received by HRQoL scores. Persons with the worst scores (< 0.00) receive more than three times greater hours of informal support than those with the best scores (0.75+). In nursing homes, those with the worst scores are more than twice as likely to have little or no participation in activities in the home and they are least likely to have a support person who is positive toward their discharge back home.

**Table 8:** Associations of global HRQoL scores with hours of informal support (home care), participation in activities and potential to return to the community (nursing homes)

HRQoL group	Home care			Nursing homes		
	*N*	Mean informal hours/week	95% CL	*N*	% Little or no participation in activities*	% with caregiver positive about return to community*
< 0.00	37,239	31.7	31.4–32.0	103,689	50.0	3.2
0.00–0.24	69,767	22.0	21.8–22.2	72,302	34.1	6.5
0.25–0.49	82,171	16.9	16.7–17.0	51,169	27.4	13.0
0.50–0.74	71,282	12.9	12.8–13.0	22,843	23.2	16.0
0.75+	47,897	9.8	9.7–9.9	6345	21.8	14.6

*P < .0001

## Discussion

The present study demonstrated that it is feasible to obtain a HRQoL measure from the interRAI assessment instruments that is crosswalked to the HUI3 standard, which is in widespread use in government surveys, clinical trials, and health economic analyses. There is clear evidence of convergent validity for the HRQoL, which had strong associations with prognosis, informal caregiver time, social participation and potential to return home from institutional settings. The interRAI HRQoL index demonstrated similar distributional properties compared to direct HUI3 measures obtained from national survey data across three distinctive populations. These findings suggest it would be appropriate to inform analyses using HUI3 to describe the general population with results from nursing home and home care settings using the interRAI HRQoL index. Given the challenges associated with selection bias and non-response for these populations in sample survey data, the interRAI data with census-level representation of eligible populations bear substantial advantages. Survey-based estimates from home care and nursing home settings are likely to be biased toward the healthiest, most cognitively intact persons in those settings who would be most able to participate and respond.

The finding that mean interRAI-measured HRQoL scores were often lower in home care clients and nursing home residents than HUI3 estimates from survey data (i.e., CCHS and NPHS) may reflect the impact of non-response bias in the survey data resulting in the exclusion of persons with substantial functional or cognitive impairments in those care settings. Since the interRAI assessments have been implemented in the majority of Canadian provinces, they represent a valuable pan-Canadian source of HRQoL data for very vulnerable populations comprised mainly of the frail elderly. Indeed, the present findings may be used as a reference standard for HRQoL scores for the province of Ontario’s home care and nursing home populations. As implementation of these instruments is completed in other provinces, it will be feasible to do inter-provincial and national comparisons.

In general, only modest differences in HRQoL scores for both the interRAI HRQoL index and HUI3 were found across age and sex groups. Similar results have been reported by Asakawa et al. [3]. For the home care and nursing home populations, this can be readily explained by the effects of eligibility criteria that would tend to make new admissions to those care settings relatively homogeneous, irrespective of demographic or diagnostic differences. That is, for younger persons to receive these services, they must demonstrate similar levels of functional impairment, medical complexity and impaired cognition, as is evident among the older service recipients. In Canada, eligibility criteria tend to be defined by function and health symptoms rather than on a demographic or diagnostic basis [7].

There are clear advantages to having an HUI3 crosswalk for the interRAI assessment instruments. Although the MDS-HSI crosswalk to HUI2 proved useful for many studies, the broader use of the HUI3 in the literature necessitated the development of the interRAI HRQoL index. Even though it was not possible to directly match every HUI3 attribute with interRAI items, the crosswalk performed similarly with respect to both the global scores and attributes.

A further advantage of the interRAI HRQoL is that it may be derived from the full suite of new interRAI assessments [17], including those for community support services, palliative care, and mental health settings. Therefore, it would be possible to examine the distribution of HRQoL across the full continuum of health services for older persons and persons with disabilities.

The key limitation to the current research is that the interRAI assessment data did not include concurrent HUI3 results for the persons assessed, so it was not possible to directly compare interRAI HRQoL and HUI3 scores at the individual level. Future research that would undertake this task should ensure that data are gathered across a variety of care settings, rather than only a single sector as was done in previous MDS-HSI research. In addition, longitudinal analyses would help to examine the degree to which the interRAI HRQoL index is responsive to change. Further, given that interRAI instruments are used internationally, it would be interesting to examine the extent to which interRAI HRQoL differs between countries and across cultures.

In summary, the present study provides evidence to support the use of the interRAI HRQoL index in vulnerable populations where global measures of HRQoL are outcomes of interest.
